# Simulating the effects of a clinical guidelines screening algorithm for fall risk in community dwelling older adults

**DOI:** 10.1007/s40520-018-1051-5

**Published:** 2018-10-19

**Authors:** Pierpaolo Palumbo, Clemens Becker, Stefania Bandinelli, Lorenzo Chiari

**Affiliations:** 1 Department of Electrical, Electronic, and Information Engineering “Guglielmo Marconi”, University of Bologna, Viale del Risorgimento, 2, 40136 Bologna, Italy; 2 Department of Clinical Gerontology, Robert Bosch Hospital, Stuttgart, Germany; 3 Geriatric Unit, Local Health Unit Tuscany Centre, Florence, Italy; 4 Health Sciences and Technologies Interdepartmental Center for Industrial Research, University of Bologna, Bologna, Italy

**Keywords:** Fall, Impact, Prevention, Risk, Validation, TUG

## Abstract

**Background:**

The current guidelines for fall prevention in community-dwelling older adults issued by the American Geriatrics Society and British Geriatrics Society (AGS/BGS) indicate an algorithm for identifying who is at increased risk of falling. The predictive accuracy of this algorithm has never been assessed, nor have the consequences that its introduction in clinical practice would bring about.

**Aims:**

To evaluate this risk screening algorithm, estimating its predictive accuracy and its potential impact.

**Methods:**

The analyses are based on 438 community-dwelling older adults, participating in the InCHIANTI study. We analysed different tests for gait and balance assessment. We compared the AGS/BGS algorithm with alternative strategies for fall prevention not based on fall risk evaluation.

**Results:**

The AGS/BGS screening algorithm (using TUG, cut-off 13.5 s) has a sensitivity for single falls of 35.8% (95% confidence interval 23.2%–52.7%) and a specificity of 84.0% (79.3%–88.4%). It marks 18.0% (13.7%–22.4%) of the older population as at high risk. A policy of targeting people with preventive intervention regardless of their individual risk could be as effective as the policy based on risk screening but at the price of intervening on 17.3% (4.1%–34.0%) more people of the older population.

**Discussion:**

This study is the first that validates and estimates the impact of the screening algorithm of these guidelines. Main limitations are related to some modelling assumptions.

**Conclusions:**

The AGS/BGS screening algorithm has low sensitivity. Nevertheless, its adoption would bring benefits with respect to policies of preventive interventions that act regardless of individual risk assessment.

**Electronic supplementary material:**

The online version of this article (10.1007/s40520-018-1051-5) contains supplementary material, which is available to authorized users.

## Introduction

Falls are common and burdensome accidents among older adults. About one-third of people over the age of 65 fall at least once a year [1]. Falls may lead to injuries and fear of falling, undermining health and wellbeing. Worldwide, it is estimated that falls are responsible for the annual loss of 35 million disability-adjusted life years [2].

Different national institutes of health have taken the guidelines for fall prevention issued by the American Geriatrics Society (AGS) and the British Geriatrics Society (BGS) [3, 4] as a model to elaborate their specific guidelines. This is the case for the National Institute for Health and Care Excellence in England and Wales [5] and the Italian National Institute of Health (Istituto Superiore di Sanità) [6].

The current AGS/BGS guidelines for fall prevention in community-dwelling older adults (last update 2011) describe a fall preventive service as composed of three main elements: a brief screening, an in-depth multifactorial fall risk assessment, and targeted interventions [3]. The screening is intended to be applied yearly on the whole population of older adults (65 years or older) to discriminate between high-risk and low-risk persons. The multifactorial fall risk assessment is then applied to those at high risk. Its function is to identify the factors underlying the fall risk and, consequently, to decide upon the most appropriate preventive intervention.

**Fig. 1:**
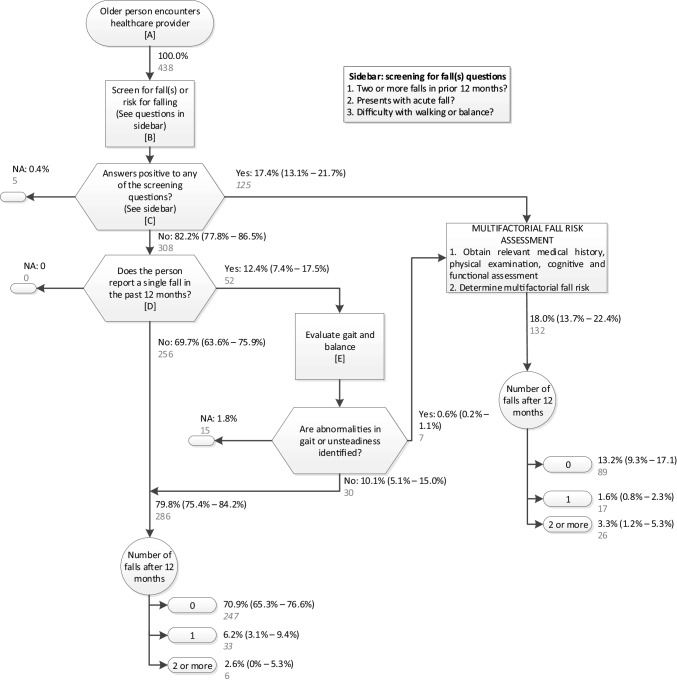
Flowchart of the AGS/BGS screening algorithm [3] and results of its application. Falls after the screening are estimated in the observational/no-preventive-intervention scenario. Letters in square brackets refer to guideline annotations. Percentages are standardized according to the distribution of age and sex in the Italian older population (as detailed in Supplementary material). 95% confidence intervals are in round brackets. Numbers of persons in the InCHIANTI dataset are in grey. *NA* data not available. Criterion for E: TUG > 13.5 s

The screening makes use of information about previous falls and difficulties or abnormalities in gait and balance. The recommendations are reported in Table S1 of the Supplementary material. The flowchart in Fig. 1 further reports the related annotations (A–E). The tests that are suggested to evaluate gait and balance (annotation E, referred in recommendations 5, 6, and 7) are the Get Up and Go test, the Timed Up And Go (TUG) test, the Berg Balance Scale, and the Performance-Oriented Mobility Assessment [3, 4].

Methodological literature about risk models in medicine has identified two important steps to evaluate a risk model before its widespread use: external validation and impact analysis. External validation is the evaluation of the predictive accuracy of the model, employing data that have not been used for its development. Impact analysis is the evaluation of the clinical and organizational consequences that are brought about by its introduction in clinical practice [7–10]. Validation of tools for fall risk assessment has also been recognized recently by the United States Preventive Service Task Force as an important research need [11].

The risk screening algorithm proposed in the current AGS/BGS guidelines has neither undergone external validation nor impact analysis. In the present study, we aim to address these two points, evaluating its predictive accuracy and estimating the effects it would have when integrated within the whole fall preventive service (i.e. when followed by preventive interventions targeted on those screened as at high risk).

## Methods

### Data

As data source, we used the InCHIANTI, an epidemiological study about mobility in the elderly [12] (ClinicalTrials.gov NCT01331512). The InCHIANTI cohort is made of subjects from Bagno a Ripoli and Greve in Chianti, two little towns in Tuscany, Italy. At baseline, persons aged 65 years or more were randomly selected from the municipality registries. People aged 90 years or more were oversampled. Other persons aged between 20 and 65 years were randomly selected and sequentially invited to ensure at least 30 men and 30 women for decade of age in this younger age group [12]. For the present study, we have used data from the 4th follow-up (FU4) of this cohort, that took place between June 2013 and July 2014. The participants received a home interview and some weeks later they were invited to a clinical centre for the clinical evaluation and the physical performance assessment (tests: TUG, Short Physical Performance Battery (SPPB) [13], and 7 m-gait test at comfortable speed). To prospectively ascertain the occurrence of falls, the participants were contacted monthly with a phone interview for the following 6 months, and with a last call 12 months after the clinical assessment. In persons who scored 18 or less in the Mini Mental State Examination (MMSE), the interviews were administered to a proxy [12].

We have simulated how the InCHIANTI participants would be classified according to the AGS/BGS screening algorithm. Occurrence and number of falls in the year prior to the assessment (needed for annotations B and D of the AGS/BGS guidelines) were directly asked to the participants during the home interview. Information about reported difficulties with walking or balance (annotation B) was derived from the question ‘Any difficulty walking across a small room?’. Assessment in gait or balance abnormalities (annotation E) was determined according to different tests. In particular, we considered TUG, SPPB, and gait speed. The case of patients presenting to the physician with an acute fall (annotation B) was not considered.

### Predictive accuracy and impact analysis

Using data about prospective falls, we have estimated fall rates for both high-risk and low-risk subjects. The true positive (TP), true negative (TN), false positive (FP), and false negative (FN) rates were calculated standardizing them to the Italian population of older adults, as explained in the Supplementary material.

The predictive accuracy was quantified from TP, TN, FP and FN in terms of sensitivity (Se), specificity (Sp), positive and negative predictive values (PPV, NPV), and accuracy (Acc).

We have taken the percentage of persons that are referred to costly multifactorial fall risk assessments and tailored interventions (Co) as a measure of the size and cost of the preventive service, and the percentage of persons that experience at least a fall in the 12 months after the screening (*F*) as a measure of its clinical effectiveness. We have assumed that the multifactorial risk assessment and the intervention determine a reduction in the risk of falling quantified by the relative risk RR = 0.78 (95% confidence interval (CI) 0.64–0.94) [14].

We have compared the advantages of the AGS/BGS screening with respect to three simple alternative scenarios that do not make use of fall risk screening: implementation of the multifactorial risk assessment and tailored intervention on none (scenario 1), everyone (scenario 2), and a fraction of the population chosen regardless of fall risk (scenario 3). For simplicity, it was assumed that the intervention-on-none scenario could be assimilated to what captured by the InCHIANTI observational data. The Co and *F* measures for all the alternatives were thus calculated as shown in Table 1 all the analyses were performed using the R statistical software, version 3.3.2 (R Core Team, Vienna, Austria) [15].

## Results

Five hundred forty-one (541) participants of the InCHIANTI FU4 survey, aged 65 years or more, received the home interview. Of those, for 438 old participants it was possible to derive information about falls during the 12-month follow-up. Table 2 gives descriptive statistics of the included sample and after standardization.

**Table 1:** Number of persons referred to multifactorial intervention (Co) and number of persons that experience at least a fall in 1 year (*F*) for the screening strategy and the three alternative scenarios

	Screening	Intervention on none	Intervention on everyone	Intervention regardless of fall risk
Co	TP + FP	0	100%	Co
*F*	RR·TP + FN	TP + FN	RR·(TP + FN)	(TP + FN) [1 − Co + Co·RR]

*TP* true positive, *FP* false positive, *FN* false negative, *RR* relative risk

**Table 2:** Descriptive statistics

	Sample (crude) statistics	Population (standardized) statistics
*N*	438	
Age	Mean (sd): 82.4 (6.5) years	Mean (sd): 75.9 (7.6) years
Gender (women)	60.7%	56.8%
MMSE	Mean (sd): 23.1 (7.9) ≥ 24: 72.3% 19–23: 8.6% 10–18: 9.6% ≤ 9: 9.6% NA: *n* = 30	Mean (sd): 25.4 (6.7) ≥ 24: 85.1% 19–23: 4.3% 10–18: 5.7% ≤ 9: 5.0%
Self-reported walking difficulties	22.8%	12.3%
Use of mobility aid	25.9% NA: *n* = 90	13.8%
TUG	Mean (sd): 12.2 (5.5) s NA: *n* = 164	Mean (sd): 10.7 (4.4) s
SPPB	Mean (sd): 8.1 (3.6) NA: *n* = 124	Mean (sd): 9.5 (3.1)
Gait speed (7 m, comfortable speed)	Mean (sd): 1.08 (0.30) m/s NA: *n* = 171	Mean (sd): 1.19 (0.28) m/s
Number of falls in the previous 12 months	0: 73.1% 1: 16.9% 2+: 10.0% NA: *n* = 7	0: 76.5% 1: 15.4% 2+: 8.1%
Number of falls in the following 12 months	0: 80.1% 1: 12.1% 2+: 7.8%	0: 85.9% 1: 8.1% 2+: 6.0%

Crude statistics are descriptive of the sample. Population statistics have been derived after standardization for age and gender according to the demographic structure of the Italian population of older adults (as mentioned in Methods and explained in Supplementary material). Descriptive statistics for the low and high risk sub-groups is given in Supplementary material

*NA* not available, *MMSE* mini mental state examination, *TUG* timed up and go test, *SPPB* short physical performance battery, *sd* standard deviation

Figure 1 shows the results of our analysis simulating the application of the screening algorithm suggested in the AGS/BGS guidelines, for the case of gait and balance assessed with TUG (cut-off 13.5 s). All the reported rates are standardized for age and sex, as detailed in the Supplementary material. 18.0% (95% CI 13.7%–22.4%) of the population would be considered at high risk and addressed with multifactorial fall risk assessment and possible multifactorial intervention, whereas 79.8% (95% CI 75.4%–84.2%) would be considered at low risk and given the sole advice to present for reassessment after 12 months. Evaluation of gait and balance with a functional test, as indicated in annotation *E*, would be required for 12.4% (95% CI 7.4%–17.5%).

**Fig. 2:**
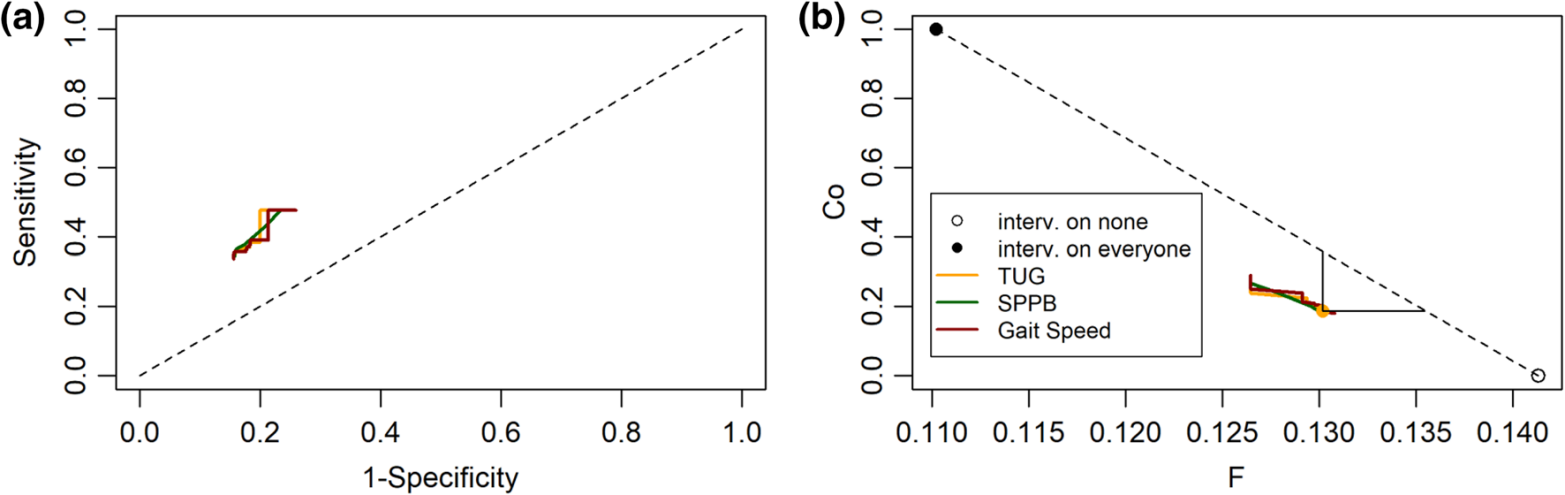
Panel a: Receiver operating characteristic (ROC) curves. Panel b: fraction of the population of older adults targeted by a preventive intervention (Co) vs fraction of the population of older adults experiencing at least one fall in the 12 months after the screening (*F*). The different colors codify different alternatives for assessing abnormalities in gait and balance: Timed Up And Go test (TUG), Short Physical Performance Battery (SPPB), and gait speed. The lines span all the possible cutoffs on these variables. Alternative preventive strategies: intervention on none (empty circle, scenario 1), intervention on everyone (filled circle, scenario 2), intervention on a given fraction of persons chosen regardless of their risk (random strategy, dashed line, scenario 3). The vertical and horizontal segments are for comparing the AGS/BGS with a cutoff of 13.5 s on TUG with the random strategy

Figure 2 reports the predictive accuracy measures in the form of Receiver Operating Characteristic (ROC) curves and the results of the impact analysis (i.e. Co and *F*), using TUG, SPPB, and gait speed for assessing gait and balance. Regardless of the functional test, the sensitivity with respect to at least one ranges from 33.8 to 47.8%, while the specificity from 74.1 to 84.4%. The extremes of these ranges do not depend on the specific functional test because they follow from assigning the whole part of the population undergoing gait and balance assessment (12.4%) to either the abnormality or non-abnormality branch of the flowchart. Tables S3 and S4 in the Supplementary material provide numerical results on particular cut-offs and for both single fallers and multiple fallers: TUG with cut-offs at 12, 13.5, and 15 s [16], SPPB with cut-offs at 9 and 11 [13], and gait speed with cut-offs at 0.8 and 1 m/s [17]. For these cut-offs the sensitivity with respect to at least one fall ranges from 35.1 to 43.3%, whereas the specificity from 79.0 to 84.3%.

In the intervention-on-none scenario (scenario 1), 14.1% (95% CI 9.8%–19%) of the older population fall at least once a year. Instead, the application of the AGS/BGS guidelines is estimated to result in 13.0% (95% CI 8.6%–17.3%) of fallers, i.e., a reduction of about 8% of fallers.

In Fig. 2b, the length of the horizontal segment indicates that, with respect to a preventive strategy that acts regardless of any fall risk estimation (scenario 3) and that reaches the same percentage of the population (18%), the application of the AGS/BGS screening algorithm would result in a further reduction of fallers equal to 0.5% (95% CI 0.08%–1.1%) of the older population.

Similarly, the length of the vertical segment indicates that the policy of targeting people regardless of their risk would be as effective as the screening policy (reduction of 8% of fallers) only at the price of intervening on 35.8%, i.e. 17.3% (95% CI 4.1%–34.0%) more people of the older population.

## Discussion

Within this study we have simulated the possible effects of the AGS/BGS screening algorithm for fall risk, evaluating its predictive accuracy and estimating its impact in a population of community dwelling older adults. The relevance of this study lies in these elements of novelty and in the important role acknowledged to the AGS/BGS guidelines for fall prevention.

We tailored the analyses according to the age and sex distribution of the Italian population of older adults. The screening would select about 18% of the older population at a high risk of falling. The sensitivity for single falls is particularly low, ranging between 35.1 and 43.3%, which implies that a high number of people would fall despite being stratified in the low risk group. The specificity is between 79.0 and 84.3%. The accuracy is higher for multiple falls than single falls, which is consistent with other results in the literature [18, 19]. Gait and balance assessment, the most time-consuming elements of the screening algorithm, would be required for only 12.4% of the persons.

Our findings also show that a hypothetical policy that addresses a given percentage of the population for prevention regardless of their risk (scenario 3) could be as effective as the screening-based policy only at the price of addressing 35.8% of the population, i.e. 17.3% more than actually needed. Although hypothetical, this random policy mimics what happens when a fall preventive service is implemented heterogeneously over the territory, without risk-related inclusion criteria.

Lamb et al. [20] tested the performance of the screening algorithm of the American Geriatrics Society/British Geriatrics Society/American Academy of Orthopaedic Surgeons (AGS/BGS/AAOS) 2001 guidelines [21] and the Ganz guidelines [22], using data from the Women’s Health and Aging Study (WHAS). This population was made up of elderly women with disabilities. For the 2001 AGS/BGS/AAOS guidelines (that are slightly different from the current ones) they reported a sensitivity between 28 and 43%, and a specificity between 74 and 88%, which are similar to our findings. In particular, the low sensitivity indicates a high false negative rate with respect to true positive rate. Instead, for the Ganz guidelines Lamb et al. reported a higher sensitivity (59%–74%) and lower specificity (42%–64%).

Recommendation number 7 of the AGS/BGS guidelines suggests that “older persons who have fallen should have an assessment of gait and balance using one of the available evaluations”. Three distinct evaluation tools have been used within the geriatric scientific community for the last two decades: the TUG [16], the SPPB [23], and gait speed [24]. In this study, we have, therefore, tested all three tools, choosing different cut-offs, and have obtained similar results. Besides considering that these measures are correlated, the similarity of results is prominently explained by the fact that, according to the guidelines, these tools have to be applied only on a part of the population which in our analysis turned out to be relatively small (12.4% of the population). While underlining the similarity of these results, we think that an analysis to appreciate the finer differences among them would require a higher sample size [in our data source only 52 older adults fell in the case of needing the assessment (Fig. 1)].

In our data source (the InCHIANTI dataset), the prospective annual incidence of falling was 19.9%. After standardizing it for age and sex, this rate further dropped to 14.1%. This rate is considerably lower than the 30% that is commonly cited (e.g. [1, 25]), but in line with other European studies [26]. It is known from the literature that fall rates are largely variable among countries and studies. The reason may lie partly in the different distribution of intrinsic risk factors among populations, and partly due to different methods employed for fall assessment [26, 27].

For simplicity, we have used the data from the InCHIANTI observational study to simulate the intervention-on-none scenario. In the Tuscany region a program of group physical exercise (the Adaptive Physical Activity program [28]) has been in place since 2006. Thus, strictly speaking, the observational scenario does not correspond to no intervention. However, in the area where the InCHIANTI study is carried out, only 2.2% of the population of older people have taken part in this program (source Regione Toscana). Therefore, we consider our assumption of no intervention under observation to be reasonable. Another assumption was to consider the relative risk (RR) of the preventive intervention equal across risk strata. This was determined by a lack of more precise evidence and is common practice for these kinds of analyses (e.g. [29]).

Preventive strategies can be population-wide or high-risk targeting. High-risk targeting strategies are needed when the preventive intervention can have harmful side effects, or when its implementation is not feasible on the whole population [30]. Traditional fall preventive interventions belong to the latter due to their cost (the harms being no greater than small [11]). Consequently, their effectiveness sensibly depends on the criteria employed for risk stratification. Nevertheless, this dependency is not usually investigated, most likely because of the difficulties associated with running large clinical trials. Our modelling approach enabled us to overcome these difficulties, carrying out an impact analysis that links the statistical predictive properties of the AGS/BGS screening algorithm, the clinical efficacy of fall preventive interventions, and the consequences in terms of number of people to intervene on and number of prevented falls.

The modest predictive accuracy of the AGS/BGS screening algorithm, and in particular its low sensitivity, suggests that developing better tools for fall prediction should be in the research agenda. Current efforts in research and innovation also strive to obtain technologies that empower elderly people and shift the preventive efforts from dedicated health centres to their homes. This shift could lead the movement in fall prevention from high-risk to more population-wide strategies.

## Conclusions

According to our analyses, the AGS/BGS screening tool for fall risk has a sensitivity for single falls between 35 and 43% and a specificity between 79 and 84%. It marks 18% of the older population as at high risk. Gait and balance assessment, that are the most time-consuming elements of the screening algorithm, are required for only 12% of the population. Despite the modest predictive accuracy of this screening, its adoption has benefits with respect to policies of preventive interventions that act regardless of individual risk assessment. These benefits could increase improving the predictive accuracy of the algorithm. The results of this study should be validated on larger observational or experimental datasets.

## Electronic supplementary material

Below is the link to the electronic supplementary material.

Supplementary material 1 (DOCX 36 KB)
