# Supplementary material for: Simulating the effects of a clinical guidelines screening algorithm for fall risk in community dwelling older adults
Source: Aging Clin Exp Res. 2018 Oct 19;31(8):1069–76. doi: 10.1007/s40520-018-1051-5 (PMC6661027; doi:10.1007/s40520-018-1051-5)
Supplement: Supplementary file 1 — Supplementary material 1 (DOCX 36 KB) [file 40520_2018_1051_MOESM1_ESM.docx]

# Simulating the effects of a clinical guidelines screening algorithm for fall risk in community dwelling older adults

Aging Clinical and Experimental Research

Pierpaolo Palumbo ^a^, Clemens Becker ^b^, Stefania Bandinelli ^c^, and Lorenzo Chiari ^a, d^

# Supplementary material

^a^ Department of Electrical, Electronic, and Information Engineering “Guglielmo Marconi”, University of Bologna, Bologna, Italy

^b^ Department of Clinical Gerontology, Robert Bosch Hospital, Stuttgart, Germany

^c^ Geriatric Unit, Local Health Unit Tuscany Centre, Florence, Italy

^d^ Health Sciences & Technologies Interdepartmental Center for Industrial Research, University of Bologna, Bologna, Italy

Corresponding author

Pierpaolo Palumbo

[pierpaolo.palumbo@unibo.it](mailto:pierpaolo.palumbo@unibo.it)

## Guidelines recommendations

Table S1. AGS/BGS recommendations for risk screening [1].

| AGS/BGS recommendations for risk screening |
| --- |
| 1. All older individuals should be asked whether they have fallen (in the past year). 2. An older person who reports a fall should be asked about the frequency and circumstances of the fall(s). 3. Older individuals should be asked if they experience difficulties with walking or balance. 4. Older persons who present for medical attention because of a fall, report recurrent falls in the past year, or report difficulties in walking or balance (with or without activity curtailment) should have a multifactorial fall risk assessment. 5. Older persons who cannot perform or perform poorly on a standardized gait and balance test should be given a multifactorial fall risk assessment. 6. Older persons who report a single fall in the past 12 months should be evaluated for gait and balance. 7. Older persons who have fallen should have an assessment of gait and balance using one of the available evaluations. 8. Older persons who have difficulty or demonstrate unsteadiness during the evaluation require a multifactorial fall risk assessment. 9. Older persons reporting only a single fall in the past year and reporting or demonstrating no difficulty or unsteadiness during the evaluation do not require a fall risk assessment. |

Among the guidelines recommendations that pertain to the screening algorithm, AGS/BGS has rated only recommendation number 7 for its strength and evidence. The level of evidence is rated as I (i.e. at least one properly done randomized controlled trial), the quality of evidence as fair (i.e. high grade evidence linked to intermediate outcome or moderate grade evidence directly linked to health outcome), and the strength of recommendation as B (i.e. “recommendation that clinicians provide this intervention to eligible patients. At least fair evidence was found that the intervention improves health outcomes and the conclusion is that benefits outweigh harm”) [1]. The evidence statement acknowledges that there has not been any prospective study that allows choosing a specific test and a specific cutoff for identifying those at greater risk of falling.

## Standardization of rates and bootstrap

In order to obtain results as indicative as possible of the application of the AGS/BGS guidelines on a national population (specifically, the Italian population), we have standardized the rates (e.g. $TP$), adjusting for the different demographic structures between the InCHIANTI cohort follow-up 4 and the Italian population of older adults.

In particular, we defined gender- and age-based strata. Age was categorized in five-year groups, from 65 onwards, grouping together all subjects aged 85 years or more. Each stratum $h$ was assigned a weight $W_{h}={N_{h}}/N$ where $N_{h}$ is the number of people in Italy at the 1^st^ of January 2017 in that stratum (as determined from the Italian National Institute for Statistics (ISTAT) [2]) and $N=\sum_{h=1}^{H} N_{h}$ is the total number of older adults.

Crude stratum-specific rates $f_{h}$ were calculated from the InCHIANTI cohort. Standardized population rates $f$ were calculated from stratum-specific rates $f_{h}$ with the so called ‘direct method’ [3]:

$f=\sum_{h=1}^{H} W_{h}f_{h}$.

Their standard errors $SE$ were calculated as

$$SE=\sqrt{\sum_{h=1}^{H} {W_{h}}^{2}\frac{f_{h}\left( 1-f_{h} \right)}{n_{h}}}$$

where $n_{h}$ is the number of persons of the InCHIANTI cohort in stratum $h$ [3].

95% confidence intervals (CI) for performance ($Se$, $Sp$, $PPV$, $NPV$, $Acc$) and impact ($Co$, $F$) measures were calculated with 1000 bootstrap resamples. For $F$, the relative risk (RR) was sampled from a lognormal distribution with 95% of its probability centered in the interval 0.64-0.94 [4].

## Missing data

For 20 samples of the InCHIANTI, we could not calculate the risk status (being considered at risk or not by the screening algorithm) because of missing data (5 had missing data on previous falls, 15 did not perform the TUG, the SPPB, or the walking test). These samples are marked as ‘NA’ in Figure 1 of the main text (annotations C and E) They represent about 4.6% of the cohort sample size (crude rate) and 2.2% of the population (adjusted rate) and were excluded from the computations of performance ($Se$, $Sp$, $PPV$, $NPV$, $Acc$) and impact ($Co$, $F$) measures.

## Descriptive statistics for sub-groups: high risk and low risk

Table S2. Descriptive statistics for sub-groups: high risk and low risk.

|  | All | | Low risk | | High risk | |
| --- | --- | --- | --- | --- | --- | --- |
|  | Sample (crude) statistics | Population (standardized) statistics | Sample (crude) statistics | Population (standardized) statistics | Sample (crude) statistics | Population (standardized) statistics |
| N, % | 438 | 100% | 286 | 79.8% | 132 | 18.0% |
| Age (mean) | 82.4 years | 75.9 years | 80.4 years | 74.1 years | 86.3 years | 83.0 years |
| Gender (women) | 60.7% | 56.8% | 53.5% | 54.7% | 74.2% | 62.8% |
| MMSE | Mean: 23.1  ≥24: 72.3%  19-23: 8.6%  10-18: 9.6%  ≤9: 9.6% | Mean: 25.4  ≥24: 85.1%  19-23: 4.3%  10-18: 5.7%  ≤9: 5.0% | Mean: 25.8  ≥24: 87.1%  19-23: 5.2%  10-18: 4.8%  ≤9: 3.0% | Mean: 26.8  ≥24: 92.6%  19-23: 1.7%  10-18: 3.9%  ≤9: 1.8% | Mean: 17.5  ≥24: 41.2%  19-23: 16.0%  10-18: 18.5%  ≤9: 24.4% | Mean: 19.9  ≥24: 55.5%  19-23: 14.1%  10-18: 12.0%  ≤9: 18.3% |
| Self-reported walking difficulties | 22.8% | 12.3% | 0% | 0% | 75.6% | 68.1% |
| Use of mobility aid | 25.9% | 13.8% | 7.5% | 2.3% | 70.3% | 62.0% |
| TUG (mean) | 12.2 s | 10.7 s | 10.6 s | 9.5 s | 19.6 s | 15.4 s |
| SPPB (mean) | 8.1 | 9.5 | 9.6 | 10.5 | 3.6 | 5.5 |
| Gait speed (7 m, comfortable speed) (mean) | 1.08 m/s | 1.19 m/s | 1.15 m/s | 1.27 m/s | 0.74 m/s | 0.89 m/s |
| Number of falls in the previous 12 months | 0: 73.1%  1: 16.9%  2+: 10.0% | 0: 76.5%  1: 15.4%  2+: 8.1% | 0: 89.5%  1: 10.5%  2+: 0% | 0: 87.4%  1: 12.6%  2+: 0% | 0: 45.4%  1: 21.5%  2+: 33.1% | 0: 35.5%  1: 19.3%  2+: 45.2% |
| Number of falls in the following 12 months | 0: 80.1%  1: 12.1%  2+: 7.8% | 0: 85.9%  1: 8.1%  2+: 6.0% | 0: 86.4%  1: 11.5%  2+: 2.1% | 0: 88.9%  1: 7.8%  2+: 3.3% | 0: 67.4%  1: 12.9%  2+: 19.7% | 0: 73.2%  1: 8.7%  2+: 18.1% |

MMSE=Mini Mental State Examination, TUG=Timed Up And Go Test, SPPB= Short Physical Performance Battery.

## Predictive performance and impact measures results

Table S3. Performance of the AGS/BGS screening algorithm according to different ways for assessing gait and balance (annotation E) and the two alternatives of intervening on none or on everyone. Performance is evaluated with respect to the outcome of at least one fall in the 12-month follow-up. Values express percentages (95% confidence interval). PPV=positive predictive value, NPV=negative predictive value, Co=fraction of people referred to multifactorial intervention, F=fraction of people that experience at least a fall in one year.

|  | **Sensitivity (%)** | **Specificity (%)** | **PPV (%)** | **NPV (%)** | **Accuracy (%)** | $\boldsymbol{Co}$ **(%)** | $\boldsymbol{F}$ **(%)** |
| --- | --- | --- | --- | --- | --- | --- | --- |
| **Screening**  (condition for gait or balance abnormality) |  |  |  |  |  |  |  |
| a - TUG > 12 s | 36.5 (22.3-54.5) | 82.5 (76.9-87.1) | 25.5 (16.8-37.6) | 88.8 (83-93.6) | 76 (70.2-81.4) | 20.2 (15.8-25.5) | 13 (8.6-17.7) |
| b - TUG > 13.5 s | 35.8 (23.2-52.7) | 84 (79.3-88.4) | 26.9 (17.3-38.8) | 88.8 (83.9-93.7) | 77.2 (72.4-82.3) | 18.8 (14.7-23.6) | 13 (8.6-17.3) |
| c - TUG > 15 s | 35.1 (22.7-52.6) | 84.1 (79.3-88.4) | 26.7 (17.5-38.7) | 88.7 (83.2-93.1) | 77.2 (71.7-82.4) | 18.6 (14.4-23.2) | 13 (9-18.2) |
| d - SPPB < 9 | 37.2 (24.1-54.1) | 83.4 (78.7-87.7) | 27 (17.8-37.6) | 89 (83.3-94) | 76.9 (71-81.8) | 19.5 (15-24.1) | 13 (8.7-18.2) |
| e - SPPB < 11 | 43.3 (28.4-62.7) | 79 (72.7-84.5) | 25.4 (16.4-35.8) | 89.4 (83.9-94.3) | 74 (67.8-79.9) | 24.2 (18.6-30.3) | 12.8 (8.5-17.8) |
| f - Gait speed < 0.8 m/s | 35.1 (22.6-52.5) | 84.3 (78.8-88.6) | 26.9 (17.7-39.1) | 88.8 (82.9-93.4) | 77.4 (71-82.5) | 18.4 (14.1-23.6) | 13 (8.9-18) |
| g - Gait speed < 1 m/s | 35.8 (22.4-54.4) | 82.4 (76.9-87.3) | 25.1 (15.9-36.5) | 88.6 (83-93.3) | 75.8 (69.8-81.5) | 20.2 (15.4-25.5) | 13 (8.9-17.7) |
| **Intervention on none** | 0 | 100 | - | 85.9 (81-90.2) | 85.9 (81-90.2) | 0 | 14.1 (9.8-19) |
| **Intervention on everyone** | 100 | 0 | 14.1 (9.9-19.3) | - | 14.1 (9.9-19.3) | 100 | 11 (7.2-15.7) |

MMSE=Mini Mental State Examination, TUG=Timed Up And Go Test, SPPB= Short Physical Performance Battery.

Table S4. Performance of the AGS/BGS screening algorithm according to different ways for assessing gait and balance (annotation E) and the two alternatives of intervening on none or on everyone. Performance is evaluated with respect to the outcome of at least two falls in the 12-month follow-up. Values express percentages (95% confidence interval). PPV=positive predictive value, NPV=negative predictive value, Co=fraction of people referred to multifactorial intervention, F=fraction of people that experience at least two falls in one year.

|  | **Sensitivity (%)** | **Specificity (%)** | **PPV (%)** | **NPV (%)** | **Accuracy (%)** | $\boldsymbol{Co}$ **(%)** | $\boldsymbol{F}$ **(%)** |
| --- | --- | --- | --- | --- | --- | --- | --- |
| **Screening**  (condition for gait or balance abnormality) |  |  |  |  |  |  |  |
| a - TUG > 12 s | 56.2 (32.2-92.8) | 82.1 (76.9-86.6) | 16.8 (8.9-27.8) | 96.7 (92.9-99.7) | 80.5 (75-85.3) | 20.2 (15.7-25.4) | 5.3 (2.4-8.7) |
| b - TUG > 13.5 s | 56.2 (27.6-89.8) | 83.6 (79.4-87.6) | 18.1 (9.7-29.2) | 96.7 (92.3-99.6) | 81.9 (76.9-86.4) | 18.8 (14.4-23.4) | 5.3 (2.5-9.2) |
| c - TUG > 15 s | 56.2 (30.3-92.6) | 83.8 (79.2-87.7) | 18.3 (10-28.6) | 96.7 (93.1-99.7) | 82.1 (76.9-86.8) | 18.6 (14.5-23.5) | 5.3 (2.5-8.6) |
| d - SPPB < 9 | 56.2 (32.1-93.2) | 82.9 (78.1-87) | 17.5 (9.3-28.4) | 96.7 (93-99.7) | 81.3 (76-85.6) | 19.5 (15.3-24.4) | 5.3 (2.5-9) |
| e - SPPB < 11 | 59 (32.3-97.4) | 78.1 (72.4-83.3) | 14.8 (8.1-24.3) | 96.7 (92.7-99.9) | 76.9 (71-82.6) | 24.2 (19.2-29.8) | 5.3 (2.4-8.6) |
| f - Gait speed < 0.8 m/s | 56.2 (30.6-91.7) | 84 (79.3-88.2) | 18.4 (9.9-29.9) | 96.7 (93.1-99.7) | 82.3 (77.2-87.2) | 18.4 (14-23) | 5.3 (2.3-8.8) |
| g - Gait speed < 1 m/s | 56.2 (30.8-92) | 82.1 (77.2-86.5) | 16.8 (8.9-27.4) | 96.7 (92.9-99.6) | 80.5 (74.9-85.3) | 20.2 (15.7-25.4) | 5.3 (2.5-8.7) |
| **Intervention on none** | 0 | 100 | - | 93.9 (90.2-97) | 93.9 (90.2-97) | 0 | 6.1 (3-9.8) |
| **Intervention on everyone** | 100 | 0 | 6.1 (3-9.5) | - | 6.1 (3-9.5) | 100 | 4.7 (2.3-7.9) |

MMSE=Mini Mental State Examination, TUG=Timed Up And Go Test, SPPB= Short Physical Performance Battery.

## References

1. Panel on Prevention of Falls in Older Persons, American Geriatrics Society and British Geriatrics Society. Prevention of Falls in Older Persons: AGS/BGS Clinical Practice Guideline [Internet]. 2011. Available from: http://geriatricscareonline.org/toc/updated-american-geriatrics-societybritish-geriatrics-society-clinical-practice-guideline-for-prevention-of-falls-in-older-persons-and-recommendations/CL014

2. Istituto nazionale di statistica. Demo Istat [Internet]. Available from: http://demo.istat.it/pop2016/index.html

3. Armitage P, Berry G, Matthews JNS. Statistical methods in medical research. 4th ed. Blackwell Science Ltd; 2002.

4. Gillespie LD, Robertson MC, Gillespie WJ, Sherrington C, Gates S, Clemson LM, et al. Interventions for preventing falls in older people living in the community. Cochrane database Syst. Rev. [Internet]. 2012;12. Available from: http://www.ncbi.nlm.nih.gov/pubmed/22972103
